# Metallohelices emulate the properties of short cationic α-helical peptides

**DOI:** 10.1039/d0sc06412b

**Published:** 2021-01-13

**Authors:** Hualong Song, Miles Postings, Peter Scott, Nicola J. Rogers

**Affiliations:** University of Warwick UK peter.scott@warwick.ac.uk nicola.rogers@warwick.ac.uk

## Abstract

Naturally occurring peptides in many living systems perform antimicrobial and anticancer host defence roles, but their potential for clinical application is limited by low metabolic stability and relatively high costs of goods. Self-assembled helical metal complexes provide an attractive synthetic platform for non-peptidic architectures that can emulate some of the properties of short cationic α-helical peptides, with tuneable charge, shape, size and amphipathicity. Correspondingly there is a growing body of evidence demonstrating that these supramolecular architectures exhibit bioactivity that emulates that of the natural systems. We review that evidence in the context of synthetic advances in the area, driven by the potential for biomedical applications. We note some design considerations for new biologically-relevant metallohelices, and give our outlook on the future of these compounds as therapeutic peptidomimetics.

## Abstract 2

Advances in metal–organic self-assembly have unearthed an area of chemical space containing peptide-emulating molecules. Comparison with natural systems indicates future directions in terms of synthesis, disease areas and mechanistic understanding.

## Graphical abstract

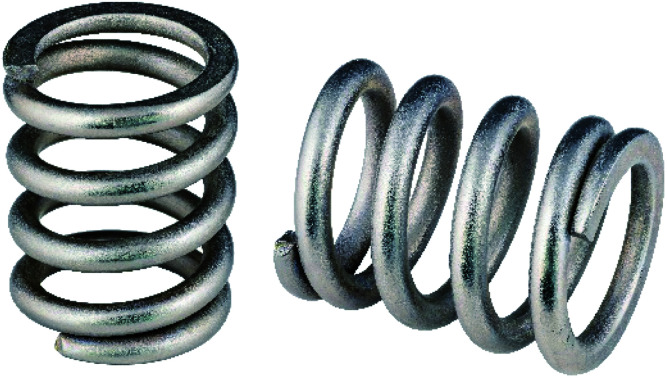

## 1. Introduction

**Fig. 1:**
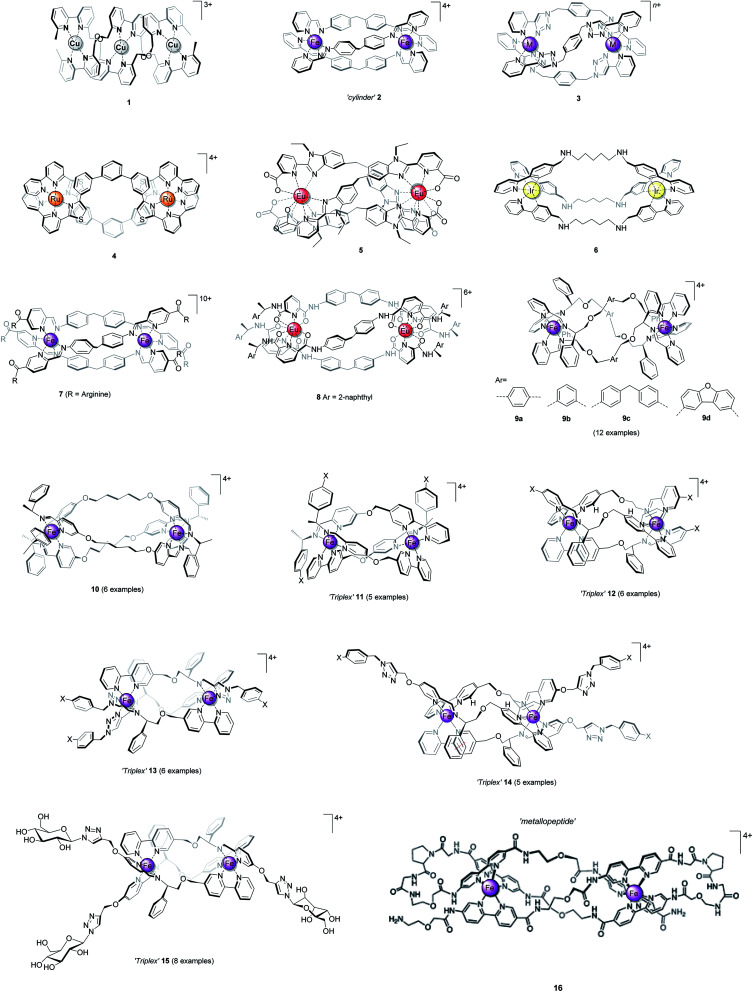
Helicate and helicate-like structures. Racemic compounds **1–6** assembled from achiral ligand strands and optically pure **7–16** assembled from ligands that incorporate elements of chirality.

In coordination complexes, the metal ions can be considered to be structural loci, providing anchoring points for the spatial distribution of coordinated ligand scaffolds, as controlled by electronic preference for particular geometries or *via* steric and secondary interactions. As nature has recognised,^1–3^ the self-assembly of such pre-programmed components gives access to architectures which are unavailable from organic chemistry.^1,4–6^ Chief among the synthetic systems are the chiral helicate structures^7–10^ (*e.g.*Fig. 1, **1–3**), formed in self-assembly processes involving two or more metal ions and a number of multi-topic ligand strands. The ligands (so-called helicands) used for this purpose must be sufficiently rigid to both avoid chelation of one ligand around a single metal, and to promote mechanical coupling between adjacent stereogenic metal coordination spheres.^10^ By this means, the absolute configuration of one metal centre is transferred to the next – the process of helication – and the self-assembled object is rendered homochiral *i.e.* all the metal centres have the same handedness Δ or Λ, leading to *P* or *M* helicity. Some closely-related structures now exist which do not rely on mechanical coupling for stereoselection, and this has enabled a number of recent breakthroughs. We refer to these latter structures as metallohelices.

It could be considered that the structures of Fig. 1 resemble the short antimicrobial α-helical peptides^11–13^ produced by all living things as part of their immune response. While the underlying chemistries of these classes of molecule are very different, they have similarities in terms of size, shape, charge, and even their amphipathic architecture. This begs the question – can we emulate the biological functions of peptide α-helices using metallohelices, particularly for applications in biomedicine?

## 2. Design considerations for metallohelices with biomedical applications

In order to interrogate this possibility, we first need to compare some fundamental properties of helicates/metallohelices with those of peptide α-helices.

If we are to deliver an intact or well-defined metal complex to *e.g.* a cellular target, it must be sufficiently soluble and stable in aqueous solutions containing various competitor ligands. Complex stability, in what would otherwise be a substitutionally labile coordination system [*e.g.* Cu(i), Fe(ii), Ln(iii)], is generally improved by the presence of chelating ligands, and moreover by a degree of preorganisation or “helication” by the strands (*vide supra*). Nevertheless, most classes of helicate for which water-soluble systems have been developed,††The majority of such architectures are charged – often cationic – but most have little solubility in water because they have weakly-coordinating counterions such as PF_6_^−^ and BF_4_^−^. This is for reasons of synthetic utility, specifically the ability to isolate the compounds by crystallisation. are insufficiently stable for the purpose of study in media or biological fluids. Rather than reversibly ‘unfolding’ like a peptide α-helix, they are irreversibly hydrolysed to sub-components. The use of kinetically inert metals [*e.g.* Ru(ii)] is commonly proposed as a solution to this problem, but syntheses using these metals almost invariably lead to kinetic mixtures of products, requiring extensive separation. For example, the Ru(ii) analogue of helicate **2** was isolated as a racemic mixture in 1% yield^14^ and **4** was synthesised with only 6% conversion to the Ru_2_L_2_ products,^15^ and this as a mixture of separable mesocates and (racemic) helicates. Lindoy,^16^ and Crowley and co-workers^17^ have successfully synthesised [Ru_2_L_3_]^4+^ helicates in much higher yields (30–60%), albeit as racemic mixtures, by employing a microwave procedure in ethylene glycol. An elegant approach to form inert racemate **3** [M = Co(iii)] was taken by Crowley and co-workers,^18^ in which bimetallic Co(ii) helicates were self-assembled under thermodynamic control, and subsequently oxidised without disruption of the architecture. Alternative approaches, including stepwise^19,20^ and preorganized^21,22^ self-assembly have also been exploited. For example Duan linked two preorganised *fac* iridium(iii) complexes, *via* reductive amination, to give the mesocate **6**.^22^ There is thus an important balance to be struck between allowing the dynamic ligand exchange processes that furnish thermodynamic control in the self-assembly process, and the stability of the final system to hydrolysis or other substitution.

Optical purity is also imperative, particularly for medicinal compounds, as living systems are rich in chiral molecules. Lehn and co-workers'^9^ prototypical double-stranded Cu(i) helicate **1** (Fig. 1) and the subsequent bimetallic triple-stranded helicates developed by *e.g.* Hannon (**2**),^23,24^ Crowley (**3**),^25^ Rice (**4**),^15^ and Bünzli (**5**)^26^ all form as racemic mixtures under thermodynamic control. Assuming sufficient solubility and stability, these mixtures must be separated in order to assess the biological activity of each enantiomer independently.^24,27,28^ For example, the resolution of Fe(ii) “cylinders” **2** (ref. 27 and 29) and their Ni(ii) analogues^24^ using cellulose stationary phase have been reported by Hannon and Qu.^24,27,28^ However, this is a cumbersome and low scale procedure, and is limited by the stability of complex whilst passing through the column.

A preferable strategy, we believe, is to incorporate elements of chirality into the system so as to drive a diastereoselective self-assembly process to a single thermodynamic product.^30,31^ While such process are ubiquitous in nature,^1^ complete chemo- and stereoselectivity is rarely achieved in synthetic systems. For helicates, some isolated studies have appeared: Hannon and co-workers grafted arginine units at the ends of the pyridylimine bidentate ligand. Control over helicity of the Fe(ii) cylinder **7** was described, within the detection limit of NMR spectroscopy^32^ although the mechanism of such stereoselection has not been addressed. Gunnlaugsson and co-workers reported the first example of stable, enantiomerically pure dimetallic lanthanide helicates; triple-stranded Eu_2_L_3_ (**8**) that displayed Eu(iii)-centred circularly polarized luminescence activity.^33^

**Fig. 2:**
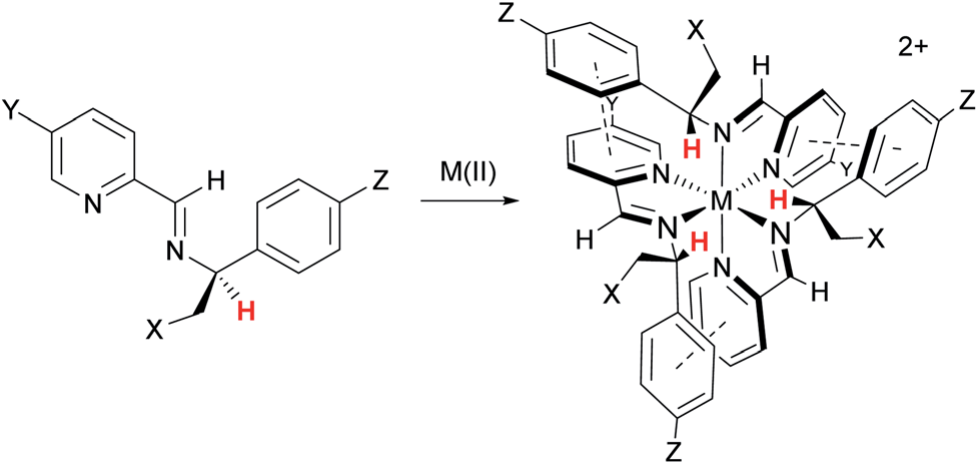
Highly diastereoselective self-assembly of *fac* tris-chelates. Pyridine-2-aldehydes and related heterocycles condense with certain α-substituted benzylamines and metals – commonly first row M(II) – to give single isomers – *fac*-Δ (shown) or *fac*-Λ depending on stereochemistry of amine – under thermodynamic control (dr > 200 : 1). This results from a combination of inter-ligand π-stacks (dashed lines) and steric effects; the benzylic H (red) is oriented toward the sterically congested region. The –CH_2_–X groups can be used to link to an adjacent tris-chelate unit forming metallohelices **9** (Fig. 1) while related strategies are used for **10–15**.

In 2009 we showed that optically pure α-methylbenzylamine and similar compounds could be used to control the absolute configuration of *monometallic* tris-chelate Fe(ii) pyridine/imine^34^ (Fig. 2) and subsequently other^35^ systems. In the case of Fe(ii), single *fac* enantiomers (dr > 200 : 1) are readily prepared by self-assembly under thermodynamic control. This allowed us to conceive of a new strategy for self-assembly of triple-stranded helicate-like bimetallics in which the absolute configuration of each metal centre is fixed independently.^36^ Freedom from the mechanical coupling paradigm allows a range of bridging units to be used – *e.g.* structures **9** which have various degrees of ‘concertina’ fold, or indeed structures like **10** (ref. 36 and 37) which have very flexible bridges. Notably, the hydrophobic π-stacking motifs in these latter structures, particularly **9**, **11**, **12**, **14**, and **15** imbue impressive resistance to hydrolysis in water and biological media, even when the efficiency of ligand folding is not high (*e.g.***9a**^36^) or the metal–ligand bonds inherently weak (**13** (ref. 38)).

α-Helices are inherently asymmetric, being formed from directional oligopeptide strands, and they typically fold into amphiphilic structures. While the metallohelices **1–10** are of rather high symmetry, simply for reasons of synthetic feasibility, some asymmetric architectures **11–16** have now emerged. The method of diastereoselection of Fig. 2 was extended to the development of what were termed ‘triplex’ metallohelices using directional heterotopic ligands. Remarkably these self-assemble with very high selectivity as asymmetric head-to-head-to-tail systems **11** and **12**; all optically pure.^36,39^ Vázquez and co-workers reported an elegant diastereoselective self-assembly approach to bimetallic asymmetric helical architectures.^40–42^ Solid-phase peptide synthesis was used to afford the carefully designed single ligand strand in self-assembling metallopeptide **16**. Bipyridine units are included for metal coordination, and two proline units create the hairpin turns and encode the sense of helical chirality.

**Fig. 3:**
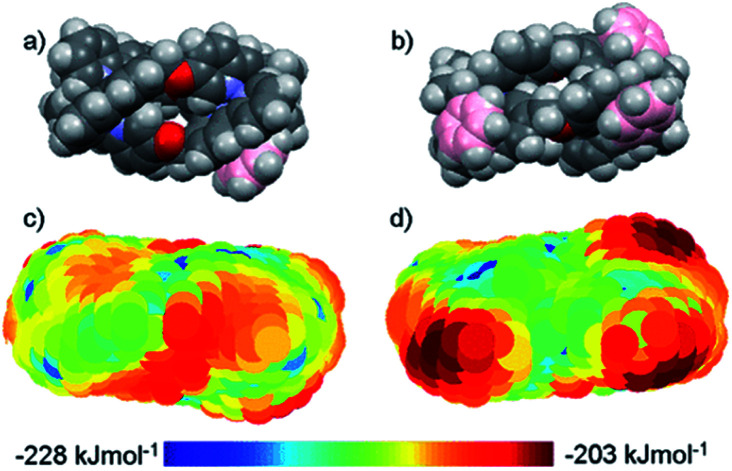
Amphipathic metallohelices. Views (a) and (b) of opposite faces of triplex metallohelix **11** and the corresponding hydrophobicity plots (c) and (d) where coloured spheres indicate the DFT-calculated positions and energies of associated water O-atoms. The most hydrophobic regions (red) correspond to the π-stacked arenes colourized pink in (a) and (b). This figure has been adapted from ref. 43 under CC BY 4.0 license from the American Chemical Society, copyright 2017.

The distinct amphiphilic character of many peptidic α-helices and related structures arises primarily from the presence of charged amino acid residues such as arginine at specific locations. In the structures of Fig. 1, notwithstanding any delocalisation to the ligands, the positive charge in most examples is merely distributed laterally, corresponding to the positions of the metal centres. In the ‘triplex’ architectures **11–15** however the asymmetric folding and the presence of charge-shielding π-stacked arenes lead to *facially* amphipathic structures comprising patchy hydrophobic regions (Fig. 3).^43^

Finally, in order for us to realistically consider biological application of any molecular system it must be synthetically achievable at a reasonable scale, and a range of structures and functionalities need to be accessible. We note that while cationic antimicrobial peptides (CAMPs) show broad-spectrum activity, with unique multi-modal mechanisms of action, the high manufacturing costs and susceptibility to proteolytic degradation have limited their pharmaceutical development.^44,45^ Systems **9–15** – all available reliably on a multi-g scale in optically pure form as a range of analogues – demonstrate that this is now becoming possible. In addition to the diversity in the core architecture it has become possible to include a range of functional groups, and these have a clear effect on biological activity. Recently, functionalized benzyl triazoles were incorporated into a triplex system (**13**).^38^ A method for the post-assembly CuAAC modification of analogues of **12** furnished with terminal alkyne groups enabled the addition of peripheral groups (*e.g.***14** (ref. 46)) allowing discovery of new functions, and the architecture is readily able to withstand addition of a library of carbohydrate units *e.g.***15**.^47^

## 3. Examples of metallohelices with biological activity

### 3.1 Biophysical studies of metallohelices with DNA and RNA

DNA is the molecular target for many chemotherapeutic ‘alkylator’ drugs, but the non-specific mode of action in which the target simply acts as a nucleophilic sink renders drugs such as cisplatin extremely toxic.^48,49^ However, the enhanced selectivity of DNA-intercalating anti-tumour antibiotics, such as doxorubicin,^50^ suggests that certain molecular events occurring on DNA – transcription, replication, repair – might be more selectively targetable than the DNA polymer itself. Therefore, disease-specific responses could be envisaged by targeting protein–DNA complexes, or DNA secondary structures.

**Fig. 4:**
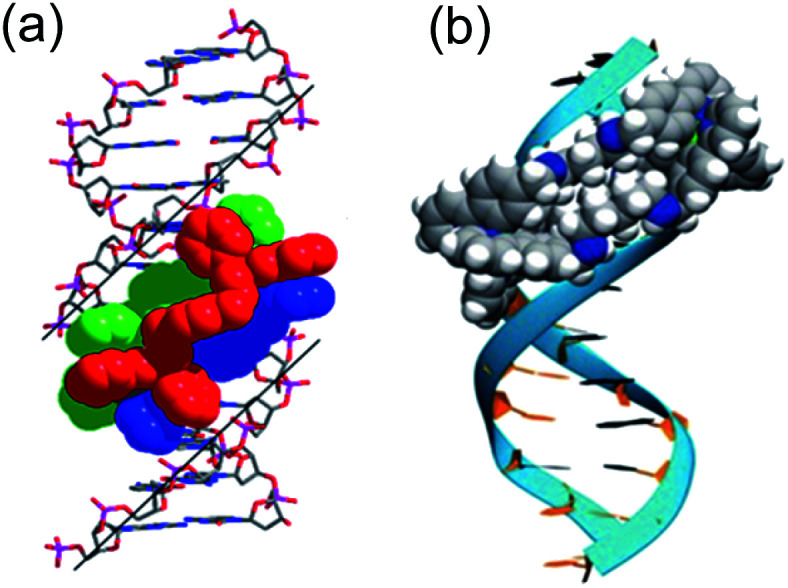
DNA-binding of metallohelices. (a) Schematic of Δ-**9a** binding to the major groove of double stranded DNA,^36^ and (b) molecular docking of *meso*-**6** binding to the minor groove of double stranded DNA.^22^ Part (b) of this figure has been adapted from ref. 22 with permission from John Wiley & Sons, copyright 2020.

Several *in vitro* studies with ‘naked’ DNA have demonstrated that various metallohelices can bind duplex DNA, since the first report by Lehn and co-workers.^51^ Apparent binding constants have been measured for several compounds *via* ethidium bromide displacement, including *P*-**2** [Fe(ii) analogue],^52^*racemic*-**3**-Co(iii),^53^*meso*-**6**,^22^ Δ-**9a**,^36^‡‡In metallohelices such as **9** the metal coordination units have the opposite helical configuration to the bridging ligand strands, so the helical descriptors *P* and *M* are unsuitable, so absolute configuration descriptors for the metal centres Δ and Λ are used. Δ-**10**,^36^ Δ-**12**,^47^ Δ-**13**,^38^ Δ-**15**;^47^ the measured *K*_app_ lie in the range 10^6^ to 10^8^, although we note the strong dependency on ionic strength. Interactions with DNA are of course anticipated for cationic complexes, simply based on electrostatic interactions, but what is more intriguing is the possibility that the unique physical dimensions and hydrophobic surfaces can complement grooves of B-DNA, akin to α-helical segments of proteins that bind to the major groove [Fig. 4(a)]. Notably, *meso*-**6** has an overall neutral charge, and still displaces cationic ethidium bromide from calf-thymus DNA, with *K*_app_ = 1 × 10^7^.^22^

Linear dichroism studies under flow conditions demonstrate that the iron cylinders **2** (and their Ru(ii) analogues)^14^ become macroscopically aligned with respect to the flow axis in the presence of DNA,^54^ indicating specifically-orientated – perhaps major groove – binding sites. Similar effects have been observed with a racemic mixture of the Co(iii) analogue of *racemic*-**3**,^53^ for **9a** [see Fig. 4(a)]^55^ and **9b**,^56,57^ the triplex metallohelices **12**,^47^**13**,^38^ and the glyococonjugates such as **15**.^47^ Molecular dynamic simulations of the interaction between B-DNA and the iron(ii) cylinder *M*-**2** were suggestive of major groove binding,^58^ whilst docking studies with mesocate **6** indicate minor groove binding [Fig. 4(b)].^22^

In contrast, analogues of **9a**/**9b** with larger bridging units (including **9c** and **9d**)^56,57^ and **10** (ref. 36) all had slightly lower apparent binding constants as indicated by ethidium bromide displacement, but no orientation with the DNA was detected by linear dichroism.

The interaction of metallohelices with more exotic DNA structures was first observed when Hannon and co-workers demonstrated that *racemic*-**2** and **7** iron cylinders induce the formation of DNA three-way junctions.^32,59,60^ Vázquez has since observed that metallopeptide **16** binds to DNA three-way junctions in preference to duplex DNA,^42^ and our collaborators have demonstrated that **9a** and **10** stabilise three-way junctions.^55^ The interactions of **9a** with both DNA and RNA ‘bulges’ have been reported,^61^ while enantiomers of **9a** both recognise and stabilise bulged RNA in the presence of duplex RNA.^61^

Brabec has recently observed that both enantiomers of **9b** can stabilise G-quadruplex DNA in the presence of excess duplex DNA,^56^ whilst Qu and co-workers have demonstrated that the iron(ii) cylinder *P*-**2**-Fe(ii), its nickel analogue *P*-**2**-Ni(ii), as well as the iron(ii) flexicate Δ-**9a** all selectively bind monomeric human telomeric G-quadruplex DNA in preference to other G-quadruplexes and duplex DNA.^24,62^ Telomeres are nucleotide sequences at the end of a chromosome, which protect its structural integrity from degradation, and are themselves protected by telomerases. Qu observed that the selectivity of the G-quadruplex binding of *P*-**2** was sensitive to the DNA loop sequence in the G-quadruplex,^63^ and that *M*-**2**, although inactive with monomeric G-quadruplexes, in fact stabilises G-quadruplex dimers.^64^ The ability to discriminate between G-quadruplexes is important for drug-targeting because the general G-quadruplex sequence motif is widespread in the human genome^65^ whereas the maintenance of human telomeric G-quadruplexes is specifically associated with tumour progression, and thus binding to these has potential anticancer activity.

Proteins involved in transcriptional regulation exploit cooperative protein–DNA interactions to enable specific DNA recognition.^66,67^ While the DNA-binding motifs often contain a major-groove binding “recognition” α-helix,^66^ other cooperative supporting contacts with the DNA backbone can be present, including additional α-helices (for example in helix-turn-helix proteins,^68^ in which an additional α-helix can bridge the major groove and the recognition α-helix), β-sheets (for example in ‘winged’ helix-turn-helix proteins),^66^ or several helical recognition elements joined together either in tandem (*e.g.* zinc-coordinating proteins^69^) or as symmetrical dimers (*e.g.* zipper-type proteins^70^). New directions for supramolecular DNA-binding entities could look towards cooperative interactions *e.g.* design strategies that induce dimers, in order to emulate the specificity of natural transcription factors. *In vitro* biophysical experiments offer insight into hypothetical mechanisms of the action of metallohelices, but since the assays are based on simplified (experimenter prescribed) systems the relevance *in vivo* is not always known. For example, while a metallohelix may interact with ‘naked’ DNA/RNA in solution, it may not be able to cross the nuclear membrane, or even if it can some other unstudied interaction may be the actual source of the biological response. That being said, a growing number of examples of biological responses to metallohelices are consistent with feasible mechanisms involving DNA-interactions, as discussed in the following sections.

### 3.2 Metallohelices with antimicrobial properties

Bacteria constitute relatively simple models for cellular life, and are an accessible starting point for assessing the bioactivity of metallohelices. Also, there is a need for new antibiotic drug candidates due to the worsening threat to global health posed by growing antimicrobial resistance, with a predicted mortality of 10 million lives per year by 2050.^66^ Antimicrobial peptides naturally occur as part of the innate immune system of plants and animals, the majority of which are the so-called cationic antimicrobial peptides (CAMPs).^71,72^ These are short peptides (10–50 amino acids long) with excess of both cationic and hydrophobic units, that often fold into secondary structures with a patchy charge distribution.^73^

As can be seen in [Table 1], several metallohelices have been tested against a range of Gram-positive and Gram-negative bacteria, using standard antimicrobial assays to determine the minimum inhibitory concentration (MIC). The antimicrobial activity is moderate for the iron(ii) cylinders *racemic*-**2**, and no activity was observed for the Ru(ii) or Co(iii) analogues of Crowley's *racemic*-**3** helicates (the MIC for the Fe(ii) racemic helicate **3** could not be attained due to poor stability in DMSO).^25^ Moderate activity was observed for compounds **10**,^37^ and interestingly no activity for the triplex systems **11**,^39^**12**,^39^ or **13**.^38^ In this context the high antimicrobial activities observed for metallohelices **9a–d** are striking.^36,56^ Further, the minimum bactericidal concentrations (MBCs) are in the range 1–2× the MIC against *E. coli* and *S. aureus*, making them by definition^69^ bactericidal. The most potent antimicrobial metallohelix reported in the literature to date – Λ-**9b** – has an MIC of 2 μg mL^−1^ against *E. coli*; which is at least as good as the antibiotic kanamycin, and its lethal effect on *E. coli* (TOP10) was observed in less than 1 h.

**Table 1:** *In vitro* antimicrobial activities (MICs) of metallohelices against Gram-positive and Gram-negative bacteria strains^a^

Compound		MIC/μg mL^−1^			Ref.
		G-positive		G-negative	
		*B. subtilis*	*S. aureus*	*E. coli*	
**2**-Fe(ii)	*Racemic*	32^a^	—	64^e^	74
**3**-Ru(ii)	*Racemic*	—	>256^b^	>256^f^	17
**3**-Co(iii)	*Racemic*	—	>1024^b^	>1024^f^	18
**9a**-Fe(ii)	Λ	**2** ^a^	**8** ^c,d^	**4** ^g,h^	36 and 56
	Δ	**1** ^a^	**8** ^c,d^	**8** ^g,h^	36 and 56
**9b**-Fe(ii)	Λ	**4** ^a^	**16** ^d^	**2** ^h^	56
	Δ	**1** ^a^	**16** ^d^	**4** ^h^	56
**9c**-Fe(ii)	Λ	**8** ^a^	**16** ^d^	64^h^	56
	Δ	**4** ^a^	**16** ^d^	32^h^	56
**9d**-Fe(ii)	Λ	**2** ^a^	**2** ^d^	**16** ^h^	56
	Δ	**2** ^a^	**2** ^d^	**16** ^h^	56
**10**-Fe(ii)	Λ	—	64^c^	32^g^	36
	Δ	—	64^c^	>128^g^	36
**11**-Fe(ii)	Λ	—	>256^b^	>256^f^	39
	Δ	—	>256^b^	>256^f^	39
**12**-Fe(ii)	Λ	—	>256^b^	>256^f^	39
	Δ	—	>256^b^	>256^f^	39
**13**-Fe(ii)	Λ	—	>128^b^	>128^f^	38
	Δ	—	>128^b^	>128^f^	38

a Bacterial strains: ^a^168, ^b^ATCC 29213, ^c^MRSA252, ^d^USA300, ^e^GM2163, ^f^ATCC 25922, ^g^MC4100, ^h^TOP10.

When applied at sub-MIC levels, Λ-**9b** induced a transcriptomic response in *E. coli* (EHEC Sakai) consistent with the expected responses to a natural CAMP, including the activation of the various two-component sensor/regulator pathways, acid response pathways and subsequent attempts by the cell to lower the net negative charge of the surface.^56^ When applied at inhibitory levels, bacterial isolates recovered were found to have only a slight increase in tolerance (MICs were only 2–4 times that of the parent strain), rather than genuine “target-site” resistance. This suggests that each of the defence responses observed in the transcriptomic studies are independently insufficient at the MIC, and rather that Λ-**9b** impacts on multiple structures or pathways that collectively kill the bacteria.

**Fig. 5:**
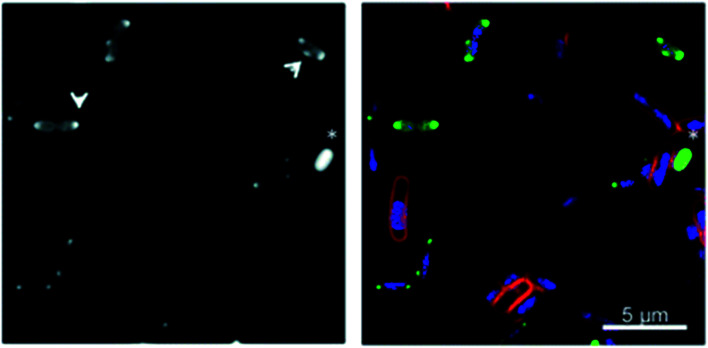
Fluorescence microscopy of metallohelices in bacteria. Adapted from ref. 56 under licence CC BY 3.0, showing sub-cellular localization of Λ-**9b′** in *E. coli* EHEC Sakai bacteria. Exponentially growing EHEC Sakai cells were treated with 8 μg mL^−1^ Λ-**9b′** and stained for membrane (FM 4-64 FX), nucleic acid (DAPI) and the Λ-**9b′** (*via* click reaction with AF-488 azide). (a) AF-488 staining (the arrows indicate the punctate localisation pattern of Λ-**9b′**, asterisk indicates an example of a bacterial cell with prominent Λ-**9b′** but lack of DAPI staining). (b) is an overlay of membrane stain (red), nucleic acid staining (blue) and ‘click’ staining of Λ-**9b′** (green).

Bacterial localisation experiments with an alkyne-labelled derivative of Λ-**9b** (Λ-**9b′**) were performed using confocal microscopy in conjunction with fluorescent “click” labelling chemistry with Alexa Fluor® 488 azide (AF-488), [Fig. 5]. Λ-**9b′** was observed to enter the cytosol of rapidly dividing bacteria, and concentrate in the polar regions of the cells.

It is possible that Λ-**9b** interacts with the microdomains of chromosome partitioning machinery known to accumulate at polar foci in cells,^56^ which would be consistent with our DNA binding studies and observations that Λ-**9b** has selective affinity for G-quadruplex DNA over double stranded DNA (see Section 3.1).

The first report of cationic antimicrobial complexes by Dwyer and co-workers appeared in the 1950s, detailing modest antimicrobial activity of relatively lipophilic Ru(ii) complexes.^75^ More recently, Collins and Keene synthesised a series of binuclear Ru(ii) complexes – related to the structures of Fig. 1 – with flexible linking bridges, and observed that longer, flexible linkers between the metal centres produced the highest activity, with MIC values of 1 μg mL^−1^ against *S. aureus* and between 2–4 μg mL^−1^ against *E. coli*, and some enantiomeric differences in potency.^76–78^ Importantly these complexes were selectively toxic against bacteria, but were non-toxic against human eukaryotic cells at concentrations much greater than the corresponding MIC value, and it has been suggested that the cellular uptake and antibacterial activity is due to their membrane-spanning ability.^79^ Solid-state NMR studies revealed that the binuclear Ru(ii) complexes incorporated into negatively charged *in vitro* models of bacteria membranes, but only associated with the surface of a charge-neutral model of a eukaryotic membrane.

Thomas and co-workers have also observed antimicrobial activity with more rigid bimetallic Ru(ii) complexes.^80,81^ The most promising lead displayed potent activity, particularly against the Gram-negative bacteria, and super-resolution luminescence microscopy revealed initial localisation of the compound at the cellular membrane, followed by localisation at the cell poles, similar to Fig. 5. Again, cell culture and animal model studies of this lead Ru(ii) complex indicate that the active complexes are not toxic to eukaryotes, even at concentrations that are several orders of magnitude higher than its MIC.

### 3.3 Metallohelices with anticancer activity

The antiproliferative activity of many metallohelices has been studied in a range of cell lines, including cancerous and non-cancerous cells. Whilst no ‘in cellulo’ model can accurately predict drug efficacy and toxicity, researchers can use these data as a yardstick to measure the relative effects of test compounds *versus* standard clinical agents, and gain insight into any fundamental selectivity differences between cancerous and non-cancerous cells.

As can be seen in Table 2, many metallohelices **9–15** demonstrate antiproliferative activity, as determined by MTT assay. In general, those tested are highly active against colon cancer cells (more so than the clinical drug cisplatin), have a range of activities against ovarian cancer cells, and show inferior activity in breast cancer cell lines.^46,53^ Duan and co-workers have recently demonstrated that the neutral Ir(iii) mesocate **6** can be used for photodynamic therapy, with activity in MCF-7 cells observed upon white light irradiation (Table 2).^22^ It is worth noting that the metallohelices exhibiting anticancer activity are all cationic, whist the charge neutral complex **6** has an IC_50_ > 30 μM without irradiation, and the neutral Ln(III) helicate **5** exhibited no effects on the cell viability of HeLa (cervical cancer) cells treated for 24 h at 500 μM (rendering these useful luminescence probes for diagnostics).^82^

**Table 2:** The antiproliferative effect (IC_50_, μM) of metallohelices on cancerous and non-cancerous cell lines^a^

Complex	Breast cancer cell lines			Ovarian cancer cell lines		Human colon cancer cell lines		Non-cancerous cell lines			Ref.
	HBL-100	MCF-7	MDA-MB-468	A2780	A2780cis	HCT116 p53^+/+^	HCT116 p53^−/−^	MRC5	ARPE-19	WI-38	
*Racemic*-**2**-Ru(ii)	*22* ^*a*^										14
*Racemic*-**2**-Fe(ii)	*27 ± 5* ^*a*^			*14 ± 2* ^*a*^				**19 ± 3** ^**a**^			32 and 86
*Racemic*-**3**-Co(iii)			*24 ± 2* ^*a*^	*7.0 ± 0.4* ^*a*^		*5.8 ± 1.7* ^*a*^		**113 ± 11** ^**a**^			53
*Racemic*-**4**						*17 ± 7* ^*b*^	**2.0 ± 0.7** ^**b**^		**>50** ^**b**^		15
*Meso*-**6**		[0.9*] ^**c**^									22
Λ-**7**				*9 ± 2* ^*a*^							32
Δ-**7**				*6 ± 3* ^*a*^							32
Λ-**9a**		*3.7 ± 0.1* ^*b*^		*4.8 ± 0.2* ^*b*^	**2.2 ± 0.1** ^**b**^	**1.7 ± 1.1** ^**b**^					55
Δ-**9a**		*3.0 ± 0.8* ^*b*^		**3.8 ± 0.1** ^**b**^	**2.4 ± 0.1** ^**b**^	**0.61 ± 0.31** ^**b**^					55
Λ-**10**		*5.5 ± 0.5* ^*b*^	*7.3 ± 0.3* ^*b*^	**3.3 ± 0.1** ^**b**^	**7.3 ± 0.3** ^**b**^	**0.62 ± 0.08** ^**b**^	**0.36 ± 0.04** ^**b**^		**7.0 ± 0.8** ^**b**^	**9.2 ± 0.8** ^**b**^	37 and 55
Δ-**10**		*10.1 ± 0.2* ^*b*^	*8.4 ± 0.4* ^*b*^	**3.48 ± 0.04** ^**b**^	*14.4 ± 0.4* ^*b*^	**0.87 ± 0.13** ^**b**^	**0.43 ± 0.06** ^**b**^		**12.0 ± 0.3** ^**b**^	**4.7 ± 0.8** ^**b**^	37 and 55
Λ-**11**			*29 ± 14* ^*b*^			**1.0 ± 0.6** ^**b**^			**8.0 ± 0.4** ^**b**^		39
Δ-**11**			*27.3 ± 4.8* ^*b*^			**2.85 ± 0.37** ^**b**^			*5.8 ± 1.8* ^*b*^		39
Λ-**12**			*7.1 ± 3.0* ^*b*^			**1.42 ± 0.39** ^**b**^			**10 ± 2** ^**b**^		39
Δ-**12**			*16.6 ± 7.8* ^*b*^	*15 ± 3* ^*a*^	*13 ± 3* ^*a*^	*21.4 ± 1.4* ^*b*^	**7.74 ± 3.68** ^**b**^		**31 ± 12** ^**b**^	**>100** ^**b**^	39 and 47
Λ-**13**						**0.19 ± 0.01** ^**b**^			*1.0 ± 0.3* ^*b*^		38
Δ-**13**		**0.15 ± 0.01** ^**a**^	**0.24 ± 0.05** ^**a**^	**0.33 ± 0.01** ^**a**^	**0.36 ± 0.02** ^**a**^	**0.32 ± 0.14** ^**b**^		*3.7 ± 0.9* ^*a*^	*6.3 ± 0.8* ^*b*^		38
Λ-**14**						**0.9 ± 0.3** ^**b**^			**8.8 ± 1.1** ^**b**^		46
Δ-**14**		*2.2 ± 0.2* ^*a*^	**2.1 ± 0.2** ^**a**^	**0.9 ± 0.2** ^**a**^	**0.24 ± 0.02** ^**a**^	**2.2 ± 1.0** ^**b**^	**3.3 ± 0.3** ^**b**^	**32 ± 5** ^**a**^	**66 ± 7** ^**b**^	**16 ± 3** ^**b**^	46
Λ-**15**						**1.99 ± 0.12** ^**b**^			**12 ± 2** ^**b**^		47
Δ-**15**				**1.4 ± 0.3** ^**a**^	**1.2 ± 0.1** ^**a**^	*6.79 ± 1.05* ^*b*^	*11 ± 2* ^*b*^		**116 ± 19** ^**b**^		47
CisPt	4.9 ± 0.3^a^^14^	1.3 ± 0.2^b^^55^	2.4 ± 0.5^b^^37^	4 ± 2^a^^32^	10.5 ± 0.2^b^^55^	3.5 ± 1.5^b^^46^	8.1 ± 1.8^b^^37^	10 ± 3^a^^38^	6.4 ± 1.0^b^^46^	2.2 ± 0.6^b^^37^	

a IC50 values in bold indicate compounds which are more active (IC^metallohelix^_50_ < IC^cisPt^_50_ in cancer cells) and/or less toxic (IC^metallohelix^_50_ > IC^cisPt^_50_ in non-cancer cells) than cisplatin. More toxic and/or less active IC_50_ values are in italics. *This is a photoactivated compound; cells were irradiated with white light (18 J cm^−2^) for 10 min followed by incubation for another 12 h. IC_50_ (dark) > 30 μM (limited by solubility). ^a^Cells were treated with complexes for 72 h; ^b^cells were treated with complexes for 96 h; ^c^cells were treated with complexes for 12 h followed by the media change, light irradiation and incubated for another 12 h.

Many of the active compounds in Table 2 also exhibit moderate/low antiproliferative activity in non-cancerous cells, and thus greater selectivity for cancer cells than cisplatin, which may be in part due to electrostatic interaction.^83–85^

For example, our metallohelices Δ-**13**, Δ-**14** and Δ-**15** have shown selectivity indices (SI, defined as IC_50_ [ARPE19]/IC_50_ [HCT116 p53^+/+^]) of *ca.* 20–30 respectively, whereas the SI of cisplatin is *ca.* 1.8.^38,46,47^ In **12–15**, enantiomeric selectivity was observed, with Λ-enantiomers often more active but less selective than their Δ analogues; Qu and co-workers have also observed significant differences between *P*-**2**-Ni(ii) and *M*-**2**-Ni(ii) (see below). No decrease in potency was observed for Δ-**9a**, Δ-**12**, Δ-**13** and Δ-**15** towards the cisplatin-resistant ovarian cancer cells (A2780cisR) compared to the cisplatin-sensitive parental cells (A2780), revealing the absence of cross-resistance and a distinct mechanism of action to that of cisplatin. Pleasingly, nearly all of the metallohelices in Table 2 (enantiomers of **10**, **11**, Δ-**12**, Δ-**14** and *racemic*-**4** (ref. 15 and 37)) are at least as active in HCT116 cells which do not express p53 (HCT116 p53^−/−^); the p53 tumour suppressor gene is one of the most frequently mutated in cancer, and is commonly ascribed to increased resistance to chemotherapeutic drugs.

We have recently tested the efficacy of the glucose-conjugated triplex metallohelix Δ-**15** in a mouse model. Following a single dose, tumour growth delays as good as cisplatin were observed, but with the advantage of no weight loss in the subjects.^47^

Despite biophysical studies demonstrating that our metallohelices **9–15** bind to ‘naked’ DNA or RNA (see Section 3.1), we have not observed any concrete evidence to date that the anticancer mechanisms of these flexicate and triplex metallohelices are associated with DNA interactions. Nuclear uptake of Δ-**12** (4.4% of total cell uptake) and Δ-**15** (13.6% of total cell uptake) has been observed in HCT116 p53^+/+^ cells,^47^ but single-cell gel electrophoresis (comet assay) analysis revealed an absence of DNA damage after the treatment of HCT116 p53^+/+^ cells with Δ-**11**, Δ-**12** and Δ-**15**.^39,47^ In addition, Δ-**12** did not induce the production of γ-H2AX (a known marker for DNA damage).^39^ Similarly, Hannon did not observe any DNA damage in HBL100 cells treated with *racemic*-**2**-Fe(ii),^86^ but Qu has observed significant induction of the phosphorylation of γ-H2AX in MCF-7 breast cancer cells and A549 lung cancer cells exposed to its nickel analogue *P*-**2**-Ni(ii) by immunofluorescence; cylinder *P*-**2**-Ni(ii) induced growth arrest in MCF-7 breast cancer cells and A549 lung cancer cells (exposed at 15 μM for several days), whereas *M*-**2**-Ni(ii) did not, despite equal uptake of both enantiomers by the cells (confirmed by mass spectrometry).^87^ Phosphorylation of γ-H2AX was not observed in primary culture of normal fibroblasts under similar conditions, suggesting some selectivity for the mode of action of *P*-**2**-Ni(ii) against cancerous cells.^88^ Intriguingly, further cell studies demonstrated that *P*-**2**-Ni(ii) induces telomere uncapping and the dissociation of telomere-binding proteins, resulting in DNA damage,^87^ which in consistent with the observed G-quadruplex binding in human telomeres *in vitro* (discussed in Section 3.1).

Given that telomerase is highly expressed in cancer stem cells (CSCs),^89,90^ there is interest in studying G-quadruplex-binding complexes in CSCs, which often cause drug resistance, tumour tissue metastasis and recurrence.^91^ Qu *et al.* reported that *P*-**2**-Ni(ii) decreased cell viability in breast CSCs (MDA-MB-231 and MCF-7) and reduced tumorigenesis of breast CSCs in mouse models.^87^ In contrast, *M*-**2**-Ni(ii) has little effect on eradicating breast CSCs. Notably, we have also observed that metallohelices Δ-**12** and Δ-**14** inhibit colonosphere formation *in vitro*, in colon CSCs (HCT116 p53^+/+^),^46^ at least as effectively than the known CSC-selective drug salinomycin.

### 3.4 Metallohelices as potential therapeutics for Alzheimer's disease

Alzheimer's disease is associated with the polymerization of amyloid-β (Aβ) peptides into extracellular amyloid fibrils,^92^ leading to abnormal build-up of insoluble proteins between brain cells. Qu and co-workers have investigated a range of metallohelices as Aβ inhibitors, and postulate that they may bind the α-helix region of Aβ peptide: a high-throughput fluorescence assay was used to show that Aβ aggregation is inhibited *in vitro* by racemic mixtures of **2**-Ni/Fe(ii),^93,94^ and by enantiomers Λ-**9a**, Δ-**9a**, Λ-**10**,^86^ Λ-**11**, Λ-**12b**, and Δ-**12b**.^95,96^ All these compounds target the α/β-discordant stretch of the peptide, and reduce Aβ cytotoxicity. The relative activities of the complexes with respect to Aβ aggregation inhibition was determined as an IC_50_ concentration, *via* a fluorescence-based screening assay as shown in Table 3.

**Table 3:** IC_50_ values of metallohelices for the inhibition of Aβ40 aggregation, measured using a high-throughput fluorescence assay^97^

Compound	IC_50_/μM	Ref.
**2**-Fe(ii) (*racemic*)	1.1	93 and 94
**2**-Ni(ii) (*racemic*)	4.6	93 and 94
Λ-**9a**	**1.7**	86
Δ-**9a**	5.4	86
Λ-**10**	6.6	86
Δ-**10**	42.4	86
Λ-**11**	3.63	37
Δ-**11**	32.3	37
Λ-**12b**	**0.94**	37
Δ-**12b**	2.55	37

In all cases the Λ enantiomers showed higher inhibition effects than the Δ enantiomers, and the more lipophilic structures were generally more effective.^37^ The most promising triplex metallohelices (Λ-**9a** and Λ-**12b**) demonstrated protective effects in PC12 (rat adrenal medulla) cells that had been exposed to cytotoxic Aβ peptides in a dose-dependent manner, and an *in vivo* study showed that Λ-**9a** and Λ-**12b** extend the life span of transgenic *C. elegans* CL2006 worms by attenuating Aβ-induced toxicity. Murine animal studies revealed that Λ-**9a** and Λ-**12b** can also cross the blood–brain barrier, which is a prerequisite for a potential therapeutic.

### 3.5 Metallohelices with anti-viral activity

The precedent for binding DNA and RNA bulges, has led to investigations into potential applications in anti-retroviral therapy; the retrovirus human immunodeficiency virus type 1 (HIV-1) contains a region of bulged RNA with high secondary structure, that complexes to the virus-encoded transactivator protein (TAT) and regulates viral transcription. Following biophysical studies that demonstrated both *M*/*P*-**2** Fe(ii) cylinders stabilise and increase the melting temperature of transactivation response region (TAR) RNA bulges, indicative of binding;^98^ studies with racemic mixtures of cylinders revealed that *racemic*-**2** Ni(ii) and *racemic*-**2** Ru(ii) inhibited HIV-1 NL4.3 replication in IG5 Jurkat T and TZM-bl HeLa cells, with limited evidence of cytotoxicity at the doses used, whilst *racemic*-**2** Fe(ii) was ineffective (notably, the activity increases in line with the stability of the metal complex). It is proposed that the cylinders bind to the bulged RNA regions, inhibiting TAT-TAR RNA complex formation, and hence block the replication. Since the replication of many RNA and DNA viruses is dependent on secondary RNA structures,^99^ and there is a growing library of metallohelical structures in the literature known to bind secondary DNA/RNA structures *in vitro* (many of which are in Fig. 1), there is potential for a new class of anti-viral agents.

## 4. Outlook

Several studies support the hypothesis that it is the physicochemical properties of short α-helical peptides that are responsible for the microbiological activity, rather than the exquisite detail of specific amino acid sequences. For example, (unnatural) l-amino acid-derived peptides are often only slightly less active against bacteria *in vitro* and have reduced susceptibility to *in vivo* proteolytic degradation.^100^ It is thus realistic that architectures with very different underlying – and perhaps more accessible – chemistries but with similar overall characteristics could be used to emulate the natural systems. This challenge has been taken up in various ways,^101^ but we note in particular Meggers' work on inert octahedral metal complexes as protein kinase inhibitors.^102^

The classes of molecule such as **9–15** have a number of useful interrelated properties. The stereogenic coordination units provided by the chemistry of Fig. 2 are controlled in large part by π-stacking. This imparts, quite unexpectedly, high stability in the complex cation such that they can be synthesised *directly* as single enantiomers in the presence of solubilising counter-ions such as chloride that might otherwise behave as competing ligands. They are also remarkably resistant to hydrolysis and able to support a wide range of peripheral functionality. Some of this synergy of properties emerges more from luck than design, and we note for example that the most benign of first row metals (iron) has exactly the right ionic radius in its divalent state for creation of optimal π-stack distance in this system, and is low-spin (diamagnetic) in this ligand environment.

The metal-based charge in these structures is delocalised to the ligands, but this is selectively shielded by the various intramolecular π-stacks leading to patchy surface hydrophobicity, the specific nature of which depends on the type of folding (Fig. 3). This is highly reminiscent of *e.g.* the cationic antimicrobial/anticancer peptides. Correspondingly, a growing number of examples of structure-dependent α-helix-like behaviours have emerged, including oriented binding to various nucleic acid structures and proteins,^32,43,55,103,104^ inhibition of ice recrystallization,^43^ and microbial cell penetration leading to a peptide-like genomic and transcriptomic response.^56^

One contrast worth noting is that while natural α-helices fold into their role-performing structure under very specific conditions, compounds like the extremely stable **9**, **11**, **12** and their derivatives are locked in a helical morphology. Also, the building blocks (sub-components) for these self-assembled architectures are relatively simple and accessible using standard organic chemistry; several examples have been made on multi-g scale. This may lead to relatively low cost of goods *cf.* peptides.

It remains however that there are rather few ways of fulfilling all the criteria outlined in Section 1. We look forward to the discovery of new platforms that deliver scalable synthesis of ranges of stable, stereochemically-pure assemblies. Some approaches hold promise, including Vasquez' elegant hybrid peptide ligand strands, and Crowley's triazole-derived systems.

While our purpose is not to emulate α-helices *per se*, rather to discover new ways to make therapeutic drugs or other useful products, a number of advances and observations described here lead us to assert that metallohelices represent an area of chemical space which is now delivering peptide-emulating molecules. Such comparison with the natural system shows us what we need to achieve synthetically, what disease areas to study, and how to interpret the outcomes of the mechanistic studies that are emerging.

## Conflicts of interest

There are no conflicts to declare.
